# Automated monitoring of brush use in dairy cattle

**DOI:** 10.1371/journal.pone.0305671

**Published:** 2024-06-25

**Authors:** Negar Sadrzadeh, Borbala Foris, Joseph Krahn, Marina A. G. von Keyserlingk, Daniel M. Weary

**Affiliations:** Animal Welfare Program, Faculty of Land and Food Systems, The University of British Columbia, Vancouver, BC, Canada; Universidade do Porto Instituto de Biologia Molecular e Celular, PORTUGAL

## Abstract

Access to brushes allows for natural scratching behaviors in cattle, especially in confined indoor settings. Cattle are motivated to use brushes, but brush use varies with multiple factors including social hierarchy and health. Brush use might serve an indicator of cow health or welfare, but practical application of these measures requires accurate and automated monitoring tools. This study describes a machine learning approach to monitor brush use by dairy cattle. We aimed to capture the daily brush use by integrating data on the rotation of a mechanical brush with data on cow identify derived from either 1) low-frequency radio frequency identification or 2) a computer vision system using fiducial markers. We found that the computer vision system outperformed the RFID system in accuracy, and that the machine learning algorithms enhanced the precision of the brush use estimates. This study presents the first description of a fiducial marker-based computer vision system for monitoring individual cattle behavior in a group setting; this approach could be applied to develop automated measures of other behaviors with the potential to better assess welfare and improve the care for farm animals.

## Introduction

Providing access to a brush promotes natural grooming behavior in cattle [1], particularly in indoor housing systems where animals have no access to trees or other natural structures to scratch [2]. Indoor housed cows are motivated to use a mechanical brush [3], and multiple factors can influence how much a brush is used by cows, including competition for access [4], cow health [5], affective state [6], and social dominance [7]. Grooming can be considered a non-essential behavior [8], so changes in brush use may provide a more sensitive indicator of health and welfare problems than measures of more essential activities like feeding and drinking [8–10].

Understanding within- and between-individual differences in brush use could facilitate the use of brushing behavior as a welfare indicator and inform brush placement decisions on dairy farms. To date there is no practical and accurate method that captures individual brush use by cattle. Brush use has been studied for research purposes using video and direct observation [11, 12], but these methods are labor intensive, limiting data collection and practicality.

Previous research has used radio frequency identification (RFID) tags tuned to the ultra-high frequency spectrum [13, 14] to identify when individual cows are within a certain distance from the brush, but these systems showed a high rate of false positives [14]. Many commercially available mechanical brushes rotate when the cow makes physical contact with it, and researchers have used this feature to improve the detection system. For instance, [13] recorded power usage of the brush to determine if the brush was on or off to reduce false positives, but despite this refinement, daily brush use detected by the device was only moderately correlated with human observations. Mandel et al. [5] developed an infrared-light based system where cow detection required rotation and motion by the brush; the resulting data were reliable, but the system required custom fitted sensors limiting its application on commercial farms.

In the quest for a more accurate and practical solution, data fusion enhanced by Machine Learning (ML) algorithms provides a promising avenue. In this approach, data from multiple sources is combined to yield more reliable outcomes [15, 16]. The integration of rotation data captured from a mechanical brush with the identification (ID) of cows in close proximity may provide a method for detecting the brushing behavior of individual cows [17]. Low frequency RFID is one possible solution for collecting animal ID as it is commonly used on dairy farms [18]. Another potential method for cow identification is via camera-aided monitoring; this approach requires less hardware and may be more practical than RFID [19, 20]. Individual identification of animals using computer vision can be aided by incorporating distinctive markers or fiducials. A fiducial marker is an object placed within the imaging system’s field of view serving as a reference point or measurement aid [21, 22]. These markers can represent data in a visual machine-readable form (e.g., barcodes and QR codes). In the current project, we used ArUco markers [23, 24], suitable for rapid, low-latency detection of 6D position estimation (3D location and 3D orientation) [24, 25]. These markers have been used to track birds [26], bees [27] and cats [28], but to our knowledge this approach has not been applied to cattle.

Our objective was to develop a system to automatically measure individual brush use by group-housed dairy cattle. Specifically, we evaluated if integrating electronically captured brush rotation data with 1) individual cow low-frequency RFID detection data, or 2) detections using a novel fiducial marker-based computer vision system, can provide an accurate estimates of daily brush use.

## Materials and methods

### Animals and housing

**Fig 1:**
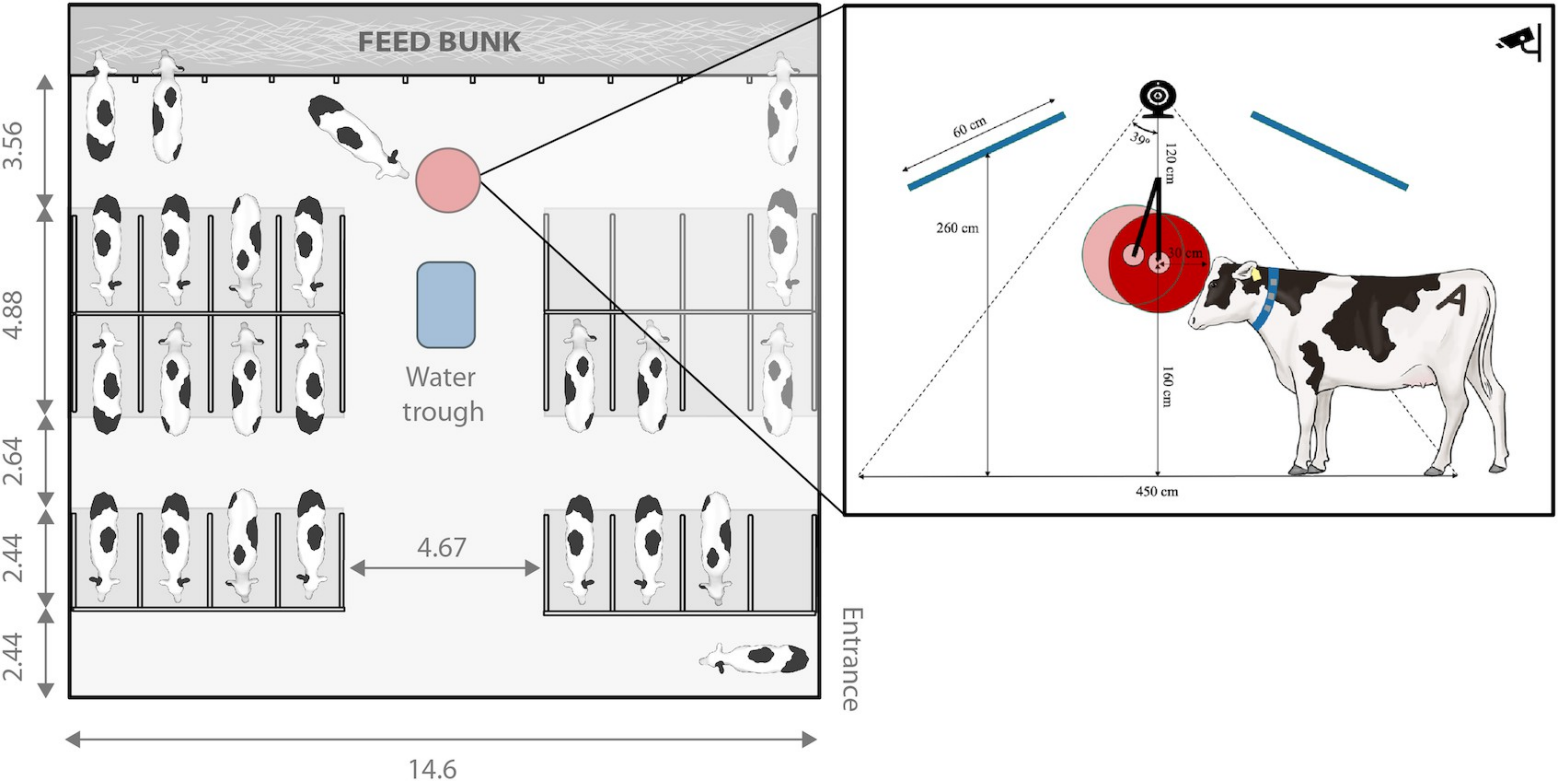
Overview of pen layout and monitoring system design. Freestall pen layout on the left, showing the location of the automated brush. Pen dimensions are shown in m. On the right, an outline of the system used to monitor cow presence near the brush (shown as red circles): two RFID antennas (shown as blue bars) and a webcam above the brush were used to detect cow ear tags and fiducial markers, respectively. A wider view of the brush area (3 m × 5 m) was captured by a CCTV camera, allowing human observers to record brush use. Illustration by Ann Sanderson (independent illustrator, Canada).

Cows were housed and cared for following the guidelines of the Canadian Council for Animal Care (CCAC 2009) and all procedures were approved by The University of British Columbia Animal Care Committee (# A19-0299). We used 24 lactating Holstein cows with an average (mean ± SD) parity of 2.4 ± 1.4, and days in milk of 256.8 ± 58.5. Cows were uniquely marked with a symbol on using hair dye and housed together in a freestall pen (16 m × 14.6 m) with 24 lying stalls bedded with sand and a post and rail feed barrier providing 0.60 m feed bunk access per cow (Fig 1). Cows were moved to the parlor twice daily at 0700 h and 1700 h for milking. They were provided *ad libitum* access to water and a total mixed ration formulated for cows in late lactation that was delivered daily at 0800 h and 1800 h.

#### Data collection

We installed a mechanical brush (LELY LUNA, Maassluis, The Netherlands) in the feeding alley opposite to the feed bunk and adjacent to the water trough. The brush was attached to a vertical arm able to swing to 90° clockwise and counterclockwise from its resting position around a single horizontal axis. As part of the design of this commercial unit, brush rotation started when the brush arm was tilted from resting position in any of two directions (as detected by two integrated light sensors), such as would occur when contacted by a cow.

We added to this commercial brush a processing unit that recorded activity associated with either of the two sensors. In this way, a new row of data was initiated every time the brush arm changed direction, which we referred to as a rotation “*event*”, recording the start time of the rotation, as well as the duration and the direction of event. These data were stored on a microSD card.

To record the ear tags of cows in proximity to the brush, we used an RFID reader (ASR650, Agrident, Barsinghausen, Germany) with two 1.0 m × 0.6 m antennas (APA160, Agrident, Barsinghausen, Germany), installed 0.8 m above the brush (as measured from the middle of the antenna and brush) at angle of approximately 30° (Fig 1). Ear tags could be detected by the antennas at a distance of up to 0.8 m, but the actual read range varied based on factors including the orientation of the tag. The antennae were connected to the processing unit of the mechanical brush and data were recorded on a microSD card.

**Fig 2:**
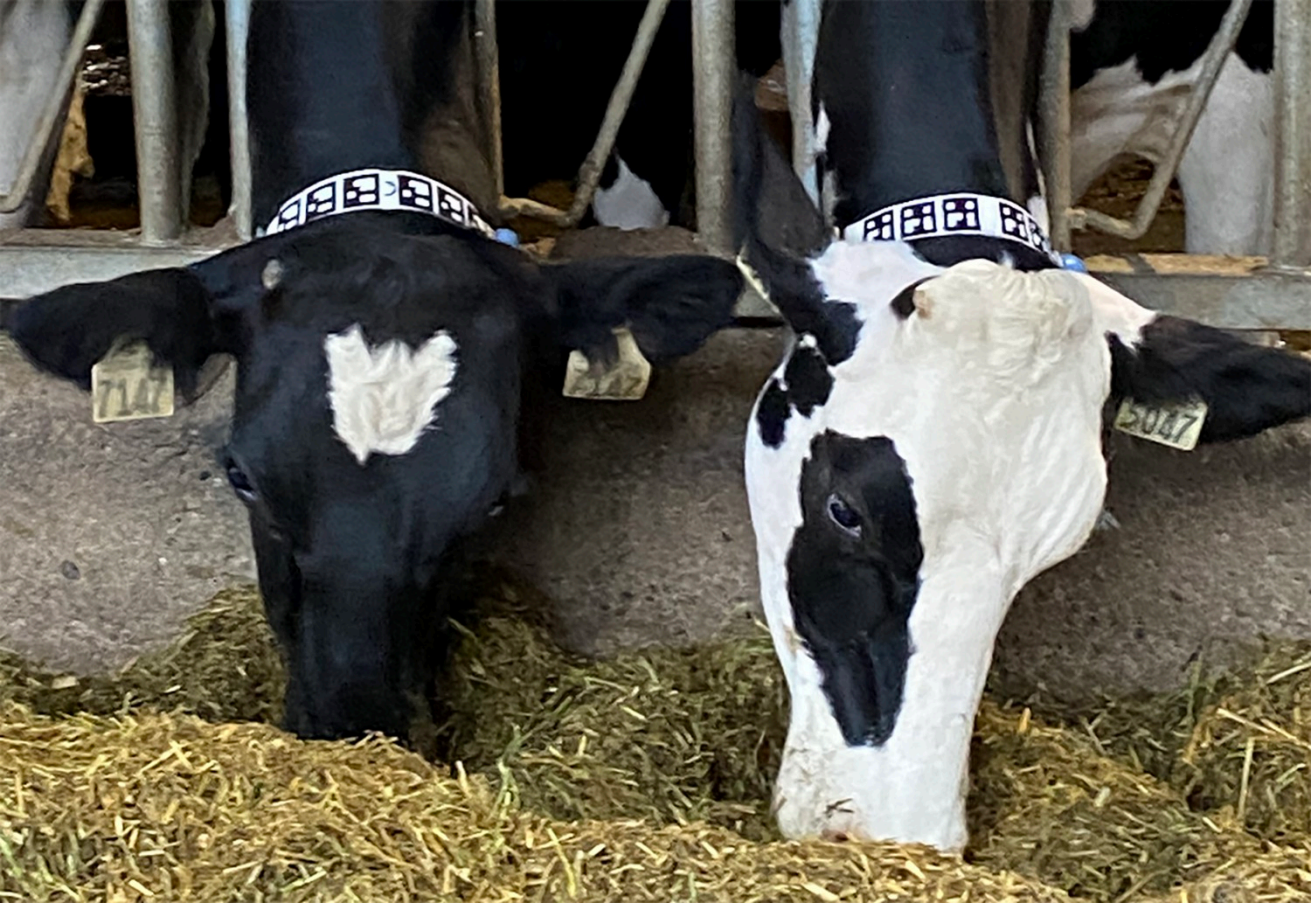
Tagged cattle. Unique ArUco tags assigned to each of the 24 cows in the group. Six identical tags were printed on Vinyl waterproof paper and attached to the collar of each cow. (Photo credit UBC Animal Welfare Program).

For vision-based identification, we used ArUco library, a popular library for generating square fiducial markers characterized by a broad black border surrounding an inner binary matrix [23, 24]. This matrix uniquely determines the marker’s identifier. We generated unique tags for each cow using ArUco’s predefined dictionary (size = 50). These tags (6.5 cm ×6.5 cm) were printed on vinyl waterproof paper and attached to the cow’s collar (Fig 2).

A webcam (ASUS Webcam C3, Taipei, Taiwan) was placed 1.2 m above the center of the brush capturing top-view footage with 78-degree field of view, a resolution of 1080 p and a frame read rate of 30 fps. A Raspberry Pie 4.0 (Raspberry Pie Foundation, Cambridge, UK), connected to the webcam and programmed with Python [29] using the OpenCV library [30], was used to detect through real-time video processing the unique fiducial markers attached to each cow when they were in the brush area (Fig 2). Data, including the time stamp, relative position of the fiducial marker in the picture, and individual cow ID, were stored on the microSD card.

We generated continuous video recordings using a CCTV camera (WV-CP310, Panasonic) placed 4 m above the brush and used 3 d of data as the ground truth for the development and validation of the proposed automated brush use detection methods. We performed continuous video observation to record cow presence in the brush area, regardless of physical contact with the brush. Each individual cow was given a distinctive mark (e.g. ‘A’) using commercially available hair dye (either black dye for while portions of the cow’s coat, or bleach for dark portions of the coat). This distinctive mark allowed observers to record individual cow identity from video.

For each instance where the cow ‘*used’* the brush, defined as direct contact with the rotating brush, or when any part of its body was obscured by the rotating brush in the video, we also captured the rotation start and end times. This information was then used to calculate the duration of brush use. The same trained human observer scored all of the video recordings, with intra-observer reliability assessed using the Intraclass Correlation Coefficient (ICC). The ICC for single events was 0.69, and for the total daily duration of brush use by individual cows, it was 0.99.

#### Data processing

We assessed individual brush use by integrating brush rotation data with cow identification data. Our analysis involved two types of brush rotation data: the ‘*events’* initially produced by the brush, and ‘*bouts’* of continuous brush use identified through machine learning (ML) techniques (referred to as bout detection).

For each rotation event or bout, we identified the user employing two strategies (user prediction): a proximity-based approach and a predictive ML model. Both user prediction strategies were applied to both the RFID and computer vision data. Human observations served as the ground truth for our analysis. We used python 3.6 [29] and Scikit-learn library [31] for developing ML models and employed cross-validated grid search to find the best hyperparameters for all models. Two days of data were used for training and one day for testing.

#### Bout detection

We used brush rotation to recognize ‘*bouts*’, defined as sequences of rotation events that corresponded to continuous usage by the same cows. To achieve this, we annotated the events as boundary or non-boundary based on human observations, where boundary events mark transitions between different users or events after more than ten seconds of brush inactivity and thus signified the start of a new bout. We used ML classification algorithms to identify boundary events by inputting sequences of events occurring before and after each event. Based on the predicted boundary events, we created bouts by grouping events occurring between two boundary events.

For each event, we determined brush inactivity duration before it occurred, labeling this as a ‘*gap’*. Each event was represented using three features: 1) gap, 2) event duration, and 3) brush displacement direction. The target output was binary, indicating whether this was a boundary event or not. Our dataset was unbalanced, with 25,564 non-boundary events and 1,035 boundary events. For training, we used only events with a gap exceeding one second, resulting in a dataset of 1,067 boundary events and 1,757 non-boundary events.

We tested and compared three commonly used supervised ML models [16, 32, 33]: 1) Random Forest (RF) [34] operates through a collection of decision trees, each providing its input on the data. By aggregating the decisions of multiple trees, it achieves a more accurate and stable prediction. It excels in scenarios with diverse variables and can handle complex classification and regression tasks. 2) Logistic Regression [35] applies a mathematical model to estimate the probability of a binary outcome, calculating the likelihood of an event (such as rain or no rain), based on several contributing factors. This model is particularly suited for either/or decisions in data. 3) Support vector machines (SVM) [36] find the optimal boundary between different data points. This method identifies the best dividing line—or hyperplane—that separates the data into classes. SVM is efficient for both linear and nonlinear datasets, making it versatile for classification problems.

#### User prediction

**Fig 3:**
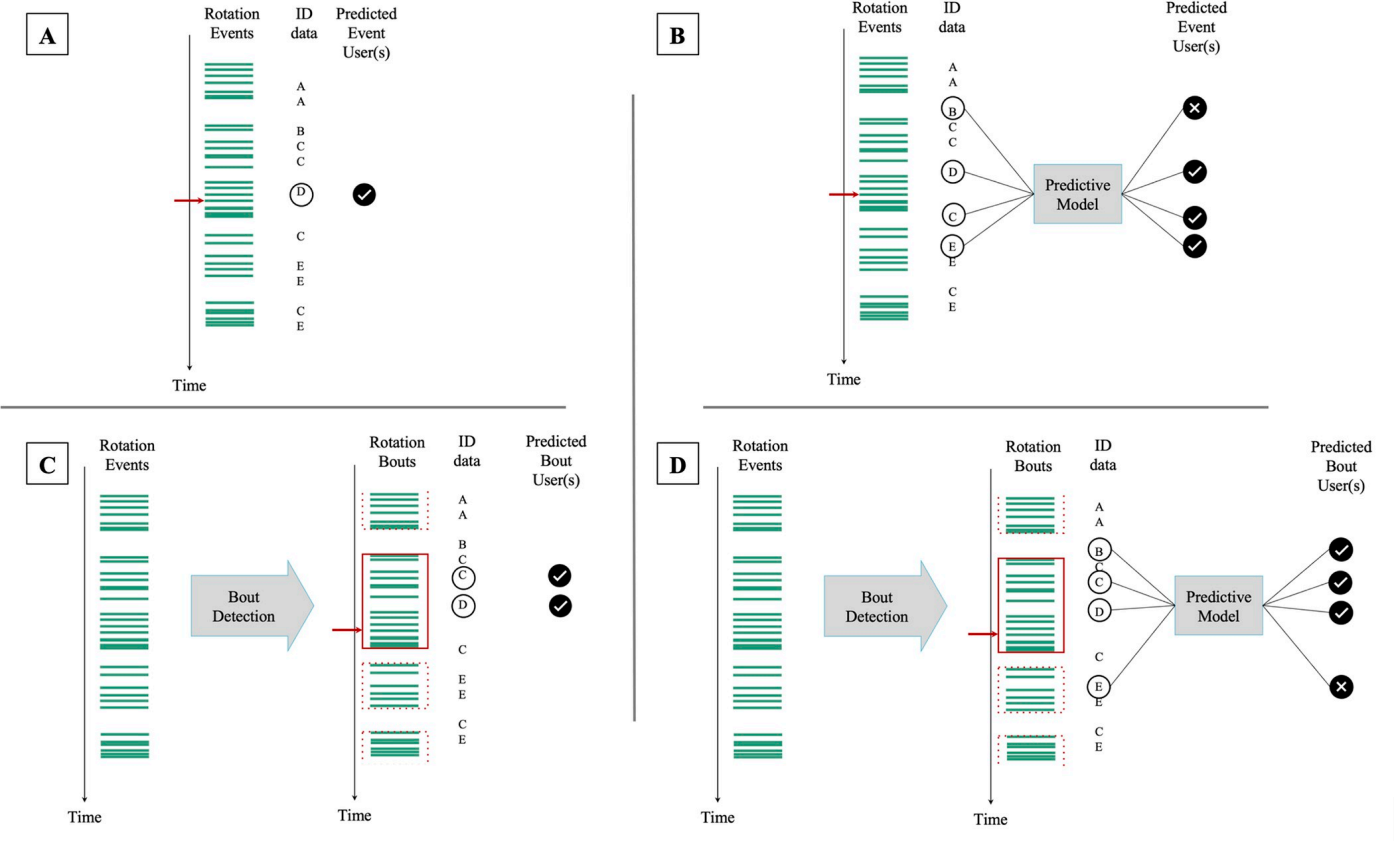
Data processing schematic. Four approaches to combine the rotation data (each green line represents a rotation event and red boxes indicate bouts (i.e., consecutive sequences of rotation events in which the brush was used by the same cows) of a mechanical brush with the identification data of cows detected by the brush (each letter represents an individual). (A, C) The "proximity" approach assigns the cow detected closest in time to the brush use event/bout as the user. (B, D) The “predictive model” approach selects the user(s) from among 4 individuals detected closest in time to each event/bout.

We employed two strategies to associate cows with specific brush rotation events or bouts, separately for the RFID and computer vision system. We annotated rotation data with the brush user’s identity, as determined by a human observer as the ground truth dataset serving as a references for developing automated brush user identification algorithms. First, we implemented a ‘*proximity*’ approach, assigning cows as brush users based on the temporal proximity of their detection to each brush rotation event/bout; the cow which was detected the closest to the event/bout was considered the user (Fig 3A–3C).

We also developed ML models (Fig 3B–3D) to identify the correct users for each brush rotation event/bout from a set of prospective users, based on their proximity in terms of detection time. We approached this as a multilabel supervised learning task, where the model input comprised the relative detection times of each prospective user to the event or bout. The output from the model was a set of binary values indicating whether each of these cows was actually using the brush at the time of the event/bout, thereby allowing for multiple identifications per event.

Given the brush’s physical dimensions and its surrounding area, it was highly improbable that more than four cows simultaneously used it or in rapid succession. Hence, we considered the four cows detected closest in time as potential users for each brush event/bout; leading to four input values for ML models and four binary outputs. We collected time-stamped observations of the four individuals detected in the brush area most temporally proximate to each rotation event or bout. We calculated the shortest time between the midpoint of the event/bout and each individual’s detection. The time intervals were coded as negative if they occurred before, zero if detection occurred during, and positive if after the event/bout. These four time-interval values formed the input features for the ML models and the binary outputs were generated based on human observation.

We had 25,564 samples of events and 278 samples of bouts. For this task we used Scikit-learn’s multi target classification which consists of fitting one classifier per target, a strategy for extending classifiers that do not support multi-target classification [31]. We evaluated 3 commonly used ML algorithms [16, 32, 33]: 1) Multi-Layer Perceptron (MLP) [37] is a kind of neural network that consists of multiple layers through which data is processed, allowing the model to learn complex patterns. It is adept at tasks where the relationship between input data and the output is intricate, making it suitable for complex classification problems. 2) Gradient Boosting [38] creates a series of models in a sequential manner, where each new model attempts to correct the errors of the previous ones. The predictions of these models are then combined to produce a final, more accurate prediction. This technique is powerful for predictive tasks where precision is key. 3) Random Forest (RF) [34] builds multiple decision trees and merges their outcomes to get more accurate and stable predictions. It’s effective because it reduces the chance of stumbling upon a single, inaccurate decision tree, as it considers the verdict of the entire ’forest’ before making a final judgment.

### Evaluation

For the bout detection, which is a single-label classification task, the evaluation metrics used were Precision, Recall, and F1-score [39]. We compared the model output to the ground truth for each data point and categorized them in one of the four groups as either a: 1) True Positives (TP), representing boundary events correctly classified as such by the model; 2) True Negatives (TN), indicating non-boundary events correctly classified as non-boundary; 3) False Positives (FP), representing non-boundary events incorrectly classified as boundary; and 4) False Negatives (FN), indicating boundary events incorrectly classified as non-boundary. Precision and recall for each model were calculated based on the distribution of the results in these categories.

$$Precision=\frac{TP}{TP+FP}$$

$$Recall=\frac{TP}{TP+FN}$$

$$F1\;score=2∙\left(\frac{Precision∙Recall}{Precision+Recall}\right)$$

For user predictions, each event or bout could be assigned to up to four cows, making this a multilabel classification task. We assessed the performance by determining the average precision and recall for the predictions of each of the four users. This approach was recommended by Tsoumakas and Katakis as effective for evaluating multi-label tasks [40]. Furthermore, a prediction containing a subset of the true labels (in this context, cows) is regarded as more accurate than one with no correct labels. To assess the similarity between the predicted and true user sets, we used the Average Jaccard Score, a metric derived from the Jaccard Index or Jaccard Similarity Coefficient [41]. Given the true set of users L_i_ and the predicted set of users P_i_ for each data point d_i_, Average Jaccard Score is calculated as:

$$Average\;Jaccard\;Score=\frac{1}{N}\sum_{i=0}^{N-1}\frac{{|P}_{i}\cap L_{i}|}{{|P}_{i}\cup L_{i}|}$$

Lastly, we computed the total daily brush use duration for individual cows, as determined through the different detection and processing methods, and compared these values with estimates from our ground truth (based upon human observations) using Pearson correlation.

## Results

For bout detection, Logistic Regression surpassed other supervised learning algorithms for classifying events as boundary versus non-boundary, in terms of achieving precision, recall, and F1-score (0.84, 0.90, and 0.87, respectively).

For user prediction, we evaluated three machine learning algorithms to identify which of the four cows detected in close temporal proximity was the actual user. MLP showed highest Average Jaccard Score when identifying incorrect users of single rotation events, for both RFID and fiducial marker data. For discerning if closely detected animals were actual users, Random Forest outperformed MLP and Gradient Boosting (Table 1).

**Table 1:** Best performing ML models for user prediction.

Identification	Rotation	ML^1^	Avg Jaccard Score	Precision	Recall	F1-score
RFID	Event^2^	MLP	0.89	0.97	0.88	0.92
	Bout^3^	RF	0.70	0.96	0.71	0.78
Fiducial Markers	Event	MLP	0.62	0.68	0.74	0.71
	Bout	RF	0.74	0.85	0.76	0.79

To identify the user of the brush during events or bouts, we evaluated three machine learning (ML) algorithms to classify four cows detected in close temporal proximity as the actual user or not. The best performing algorithm, primarily assessed using the Average Jaccard Score, varied depending on the identification context (i.e. events vs. bouts), and the detection method used (i.e. RFID vs. computer vision with fiducial markers).

^1^ The machine learning algorithm that achieved the highest Average Jaccard Score among Multilayer Perceptron (MLP), Gradient Boosting, and Random Forest (RF) models tested.

^2^ Rows of rotation data initially produced by the brush^.^

^3^ Sequences of rotation events that corresponded to continuous usage by the same cows.

**Fig 4:**
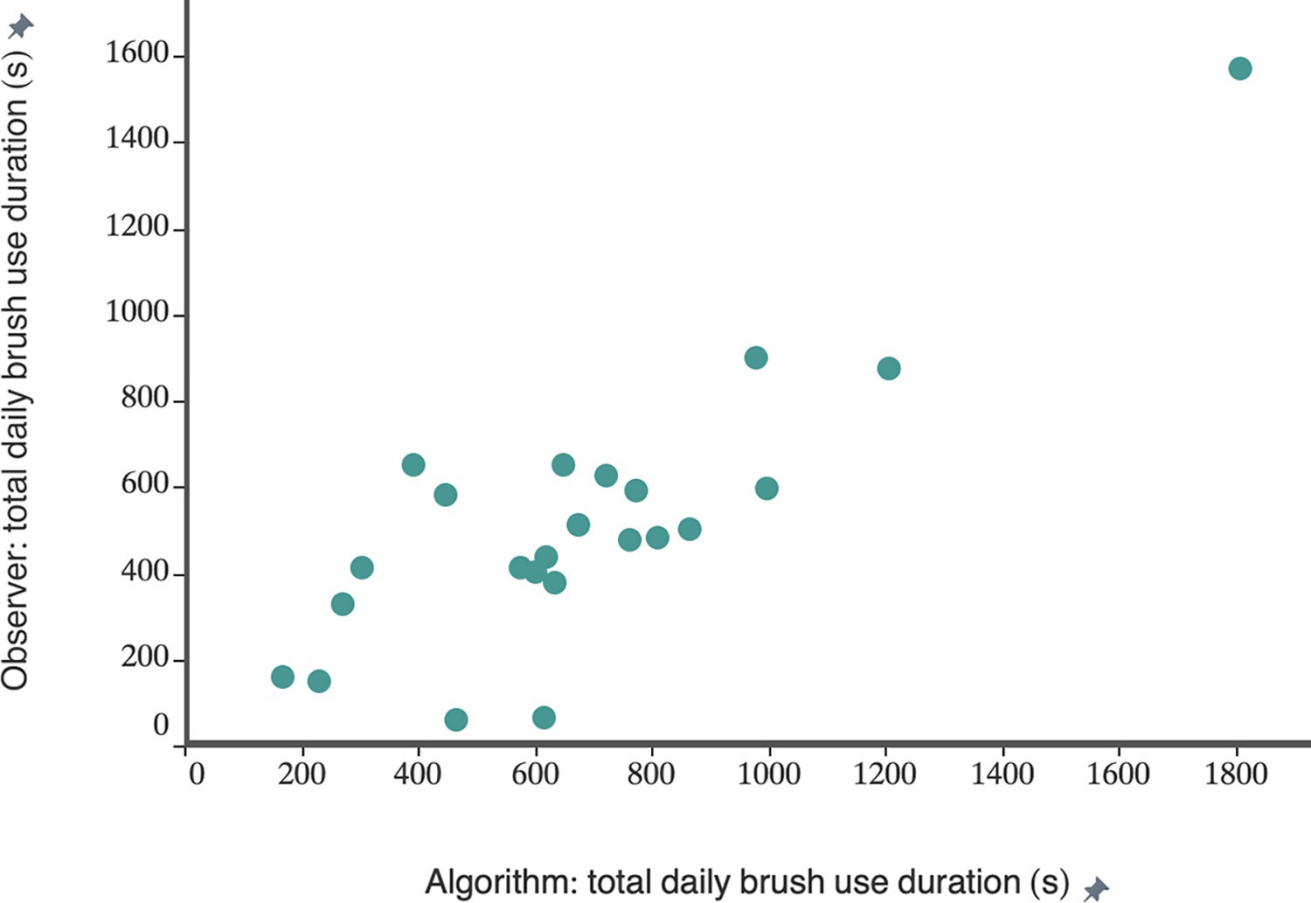
Daily brush use estimation: Observer vs. automated method. Correlation between the total daily brush use duration of 24 lactating dairy cows as determined by human video observation and an algorithm combining brush rotation data and cow detections based on a computer vision system with fiducial markers and using proximity method for integration (r = 0.84).

We calculated the total daily brush use duration for individual cows using the best performing ML algorithm to assign cows to brush rotation times based on all proposed approaches (Fig 3); the resulting values were then compared with our ground truth estimates for total daily brush use. According to human video observation, the total daily brush use ranged between 59 and 1610 s (Fig 4). Table 2 shows the correlation between total daily brush use of individual cows determined by the human observer and the different automated approaches used to estimate individual brush use. Estimates based upon brush rotation bouts combined with fiducial marker detection using the “proximity” method showed the strongest association with ground truth data (r = 0.84, Fig 4) followed by estimates based on brush rotation events combined with fiducial marker detection using ML for user prediction (r = 0.81), achieved by Multi-Layer Perceptron algorithm (Table 1).

**Table 2:** Comparison of different approaches based on daily brush use estimation.

Identification	Bout/Event	Integration	Correlation
RFID	Event^1^	Proximity^3^	0.32
		ML^4^	0.44
	Bout^2^	Proximity	0.43
		ML	0.47
Fiducial Markers	Event	Proximity	0.75
		ML	0.81
	Bout	Proximity	0.84
		ML	0.79

Pearson correlation coefficients between total daily brush use of cows (n = 24) as estimated by human video observation (ground truth) versus different automated detection algorithms based on either events, or bouts of brush rotation data, and either radio frequency identification (RFID) or computer-vision detection using fiducial markers.

^1^ Rows of rotation data initially produced by the brush.

^2^ Sequences of rotation events that corresponded to continuous usage by the same cows.

^3^ The "proximity" approach assigns the individuals detected closest in time as the user(s).

^4^ The “predictive ML” approach, uses machine learning to select the user(s) from among individuals detected closest in time.

## Discussion

Our study developed and evaluated two methods for detecting individual brush use by freestall group-housed dairy cattle provided access to a rotating mechanical brush. We assessed the feasibility of integrating brush rotation data with cow identification data acquired using RFID or a novel fiducial marker-based computer vision system. In both cases we applied ML algorithms to estimate individual brush use.

### Detection of cows via computer vision compared to RFID

Although both approaches were able to detect individual cows in proximity to the brush, the computer vision-based system did so with higher accuracy in measurement of the total daily brush use. In some ways the RFID system benefited from a simplified initial setup, as cows were already equipped with RFID ear tags for identification, but fitting the brush with antennae required some investment in time and equipment. The poorer performance of the RFID-based system was associated with an overall lower number of cow detections compared to the computer vision approach. Detection issues can be caused by multiple factors common such as 1) metal and water interference, 2) orientation of tags and antennae, and 3) presence of more than one animal. Low frequency RFID systems are susceptible to interference caused by the presence of metal, which is commonly found in dairy farm environments [42]. This interference can result in reduced signal strength and detection accuracy. Due to the limited reading distance for low-frequency RFID (0–0.80 m), the RFID system relies on the orientation and number of antennas; others have also reported challenges with the accuracy and limited reading range of this system [42–44]. Despite our best attempt to tune the system, the orientation of the RFID tags and antennas may not have been optimal, potentially explaining the lower performance compared to the computer vision-based system. Jebali & Kouki [45] showed that the orientation of the ear tag can affect the reading. In their study, ear tags were not detected when in 90-degree orientation. In addition, RFID systems are limited in detecting multiple animals, as the signals from different RFID tags may overlap [17, 46].

Our computer vision approach allowed for more accurate estimates of brush use compared to the RFID-based system. This method involved attaching fiducial markers to the collars of cows and required only a webcam and a relatively simple processor. Our results indicate the potential of computer vision-based approaches using fiducial markers as a cost-effective and applicable solution for monitoring cow behavior. However, computer vision systems are not without limitations; factors like uneven lighting, tag occlusion, camera cleanliness, and tag cleanliness can impact performance [24, 47]. While some of these challenges can be mitigated using advanced image processing techniques, others remain intrinsic to the system. For instance, the system might fail to record cows where tags are not visible (i.e., when the neck is under the brush) or when dirt, mud, or debris obstruct the fiducial markers or the camera lens—common occurrences in dairy barns.

Both identification methods are dependent upon the cow’s head being in close proximity to the brush. Events where cattle engage the brush with the back part of their body present an identification challenge, leading to potential misattribution of brush use. We propose the exploration of a more extensive monitoring area and the integration of biometric-based identification to mitigate identification and data attribution discrepancies.

### ML improved accuracy of brush use measurement

While integrating the identification data with the rotation data, we distinguished between two levels of detail: singular rotation events and extended rotation sequences termed bouts. By utilizing machine learning (ML) to identify the onset of rotation sequences, and attributing these bouts to specific cows, we were able to enhance the precision of our brush usage estimates compared to the original events generated by the brush.

Employing ML algorithms to merge individual cow detection with brush rotation events yielded more precise assessments of brush utilization for each cow, outperforming the basic "proximity" approach. For bout-based approaches, employing machine learning slightly improved the accuracy of measuring overall brush use when using RFID identification. However, it did not enhance the accuracy of measuring overall brush usage with the fiducial marker identification method. This discrepancy may be attributed to the limited number of bout examples available for ML algorithm training compared to the more abundant event samples. We hypothesize that increasing the dataset with more bout examples could improve the results. Additionally, while we evaluated some commonly used ML algorithms, exploring other algorithms that may be better suited to this specific problem could further improve accuracy.

The strong correlations between total daily brush use duration as estimated by human observers and the machine learning-based methods suggest that automated methods can provide accurate estimates of individual brush use in group housed dairy cattle. However, real-world application and scalability of these systems should be considered. In our study a small group of lactating dairy cows was used, and individual brush use ranged from a few minutes to almost half an hour a day. Housing conditions, brush location, group size, and the age or production status of the animals may impact brushing behavior and the accuracy of our proposed methods. Evaluating performance in diverse environmental conditions and varied cattle populations would provide insights into the robustness of these methods. An additional point to consider when developing systems such as the one described here is understanding how the animal’s behaviors are captured by different detection methods. Additional refinement of the machine learning algorithms would also allow for enhancements in accuracy and reliability in diverse settings.

## Conclusions

A system integrating brush rotation data with either RFID or computer vision-based cow identification, and processed utilizing machine learning algorithms, can effectively detect individual brush use in group-housed dairy cattle. The ability to automate collection of individual brush use data may prove useful for future work focused on understanding how automated brushes should be used on farms (including how many are required and where they should be best placed) and in developing tools for monitoring cow health and welfare on dairy farms. Our results provide the first evidence that a fiducial marker-based computer vision system can be used to monitor cow presence in dairy barns; we suggest that other researchers consider this approach in developing methods for farm animal monitoring.

## References

[1] Wilson SC, Mitlöhner FM, Morrow-Tesch J, Dailey JW, McGlone JJ. An assessment of several potential enrichment devices for feedlot cattle. Appl Anim Behav Sci. 2002;76: 259–265. doi: 10.1016/S0168-1591(02)00019-9

[2] DeVries TJ, Vankova M, Veira DM, Von Keyserlingk MAG. Short Communication: Usage of Mechanical Brushes by Lactating Dairy Cows. J Dairy Sci. 2007;90: 2241–2245. doi: 10.3168/JDS.2006-648 17430923

[3] McConnachie E, Smid AMC, Thompson AJ, Weary DM, Gaworski MA, Von Keyserlingk MAG. Cows are highly motivated to access a grooming substrate. Biol Lett. 2018;14. doi: 10.1098/RSBL.2018.0303 30089661 PMC6127119

[4] Val-Laillet D, Veira DM, Von Keyserlingk MAG. Short Communication: Dominance in Free-Stall—Housed Dairy Cattle Is Dependent upon Resource. J Dairy Sci. 2008;91: 3922–3926. doi: 10.3168/JDS.2008-1332 18832215

[5] Mandel R, Nicol C, Whay H, Klement E. Short communication: Detection and monitoring of metritis in dairy cows using an automated grooming device. 2017. doi: 10.3168/jds.2016-12201 28478012

[6] Lecorps B, Welk A, Weary DM, von Keyserlingk MAG. Postpartum Stressors Cause a Reduction in Mechanical Brush Use in Dairy Cows. Animals 2021, Vol 11, Page 3031. 2021;11: 3031. doi: 10.3390/ANI11113031 34827764 PMC8614528

[7] Foris B, Lecorps B, Krahn J, Weary DM, von Keyserlingk MAG. The effects of cow dominance on the use of a mechanical brush. Scientific Reports 2021 11:1. 2021;11: 1–7. doi: 10.1038/s41598-021-02283-2 34837005 PMC8626463

[8] Hart BL. Biological basis of the behavior of sick animals. Neurosci Biobehav Rev. 1988;12: 123–137. doi: 10.1016/S0149-7634(88)80004-6 3050629

[9] Spruijt BM, Van Hooff JARAM, Gispen WH. Ethology and neurobiology of grooming behavior. Physiol Rev. 1992;72: 825–852. doi: 10.1152/physrev.1992.72.3.825 1320764

[10] Mattiello S, Battini M, De Rosa G, Napolitano F, Dwyer C. How Can We Assess Positive Welfare in Ruminants? Animals (Basel). 2019;9: 758. doi: 10.3390/ANI9100758 31581658 PMC6826499

[11] Proudfoot K, Reyes FS, Gimenez AR, Anderson KM, Miller-Cushon EK, Dorea JR, et al. Impact of Stationary Brush Quantity on Brush Use in Group-Housed Dairy Heifers. Animals 2022, Vol 12, Page 972. 2022;12: 972. doi: 10.3390/ANI12080972 35454219 PMC9027817

[12] Horvath KC, Allen AN, Miller-Cushon EK. Effects of access to stationary brushes and chopped hay on behavior and performance of individually housed dairy calves. J Dairy Sci. 2020;103: 8421–8432. doi: 10.3168/JDS.2019-18042 32564951

[13] Falk M, Cantor MC, Hayes M, Jackson J, Costa JC. Validation of radio frequency identification with a current transducer to quantify the use of an automatic grooming brush in pre-weaned dairy calves. 10th International Livestock Environment Symposium, ILES 2018. 2018. doi: 10.13031/iles.ILES18-107

[14] Toaff-Rosenstein RL, Velez M, Tucker CB. Technical note: Use of an automated grooming brush by heifers and potential for radiofrequency identification-based measurements of this behavior. J Dairy Sci. 2017;100: 8430–8437. doi: 10.3168/JDS.2017-12984 28803017

[15] Mitchell HB. Data fusion: Concepts and ideas. Data Fusion: Concepts and Ideas. 2012. doi: 10.1007/978-3-642-27222-6

[16] Meng T, Jing X, Yan Z, Pedrycz W. A survey on machine learning for data fusion. Information Fusion. 2020;57: 115–129. doi: 10.1016/J.INFFUS.2019.12.001

[17] Ntafis V, Patrikakis CZ, Fragkiadaki EG, Xylouri-Fragkiadaki EM. RFID Application in Animal Monitoring. The Internet of Things. 2008; 165–184. doi: 10.1201/9781420052824-8

[18] Voulodimos AS, Patrikakis CZ, Sideridis AB, Ntafis VA, Xylouri EM. A complete farm management system based on animal identification using RFID technology. Comput Electron Agric. 2010;70: 380–388. doi: 10.1016/J.COMPAG.2009.07.009

[19] Porto SMC, Arcidiacono C, Anguzza U, Cascone G. The automatic detection of dairy cow feeding and standing behaviours in free-stall barns by a computer vision-based system. Biosyst Eng. 2015;133: 46–55. doi: 10.1016/J.BIOSYSTEMSENG.2015.02.012

[20] Nasirahmadi A, Edwards SA, Sturm B. Implementation of machine vision for detecting behaviour of cattle and pigs. Livest Sci. 2017;202: 25–38. doi: 10.1016/J.LIVSCI.2017.05.014

[21] Fiala M. Designing highly reliable fiducial markers. IEEE Trans Pattern Anal Mach Intell. 2010;32: 1317–1324. doi: 10.1109/TPAMI.2009.146 20489233

[22] Atcheson B, Heide F, Heidrich W. CALTag: High Precision Fiducial Markers for Camera Calibration. 2010. doi: 10.2312/PE/VMV/VMV10/041-048

[23] Romero-Ramirez FJ, Muñoz-Salinas R, Medina-Carnicer R. Speeded up detection of squared fiducial markers. Image Vis Comput. 2018;76: 38–47. doi: 10.1016/J.IMAVIS.2018.05.004

[24] Garrido-Jurado S, Muñoz-Salinas R, Madrid-Cuevas FJ, Marín-Jiménez MJ. Automatic generation and detection of highly reliable fiducial markers under occlusion. Pattern Recognit. 2014;47: 2280–2292. doi: 10.1016/J.PATCOG.2014.01.005

[25] Lightbody P, Krajník T, Hanheide M. An efficient visual fiducial localisation system. ACM Sigapp Applied Computing Review. 2017;17: 28–37. doi: 10.1145/3161534.3161537

[26] Alarcón-Nieto G, Graving JM, Klarevas-Irby JA, Maldonado-Chaparro AA, Mueller I, Farine DR. An automated barcode tracking system for behavioural studies in birds. Methods Ecol Evol. 2018;9: 1536–1547. doi: 10.1111/2041-210X.13005

[27] Crall JD, Gravish N, Mountcastle AM, Combes SA. BEEtag: A low-cost, image-based tracking system for the study of animal behavior and locomotion. PLoS One. 2015;10. doi: 10.1371/JOURNAL.PONE.0136487 26332211 PMC4558030

[28] Eagan BH, Eagan B, Protopopova A. Behaviour Real-Time Spatial Tracking Identification (BeRSTID) used for Cat Behaviour Monitoring in an Animal Shelter. Scientific Reports 2022 12:1. 2022;12: 1–9. doi: 10.1038/s41598-022-22167-3 36266417 PMC9584257

[29] Van Rossum G L. Drake F Python 3 Reference Manual, Scotts Valley. Scotts Valley, CA. 2009.

[30] Bradski G. The OpenCV Library. Dr Dobb’s Journal of Software Tools. 2000.

[31] Pedregosa F, Varoquaux G, Gramfort A, Michel V, Thirion B, Grisel O, et al. Scikit-learn: Machine learning in Python. Journal of Machine Learning Research. 2011;12: 2825–2830.

[32] Aguilar-Lazcano CA, Espinosa-Curiel IE, Ríos-Martínez JA, Madera-Ramírez FA, Pérez-Espinosa H. Machine Learning-Based Sensor Data Fusion for Animal Monitoring: Scoping Review. Sensors 2023, Vol 23, Page 5732. 2023;23: 5732. doi: 10.3390/S23125732 37420896 PMC10305307

[33] Lürig MD, Donoughe S, Svensson EI, Porto A, Tsuboi M. Computer Vision, Machine Learning, and the Promise of Phenomics in Ecology and Evolutionary Biology. Front Ecol Evol. 2021;9: 642774. doi: 10.3389/FEVO.2021.642774/BIBTEX

[34] Breiman L. Random forests. Mach Learn. 2001;45: 5–32. doi: 10.1023/A:1010933404324/METRICS

[35] Hosmer DW, Lemeshow S. Applied logistic regression. 2nd Edition. John Wiley & Sons, Inc. 2000.

[36] Cortes C, Vapnik V, Saitta L. Support-vector networks. Machine Learning 1995 20:3. 1995;20: 273–297. doi: 10.1007/BF00994018

[37] Goodfellow lan Bengio Yoshua CA. Deep Learning—Ian Goodfellow, Yoshua Bengio, Aaron Courville—Google Books. MIT Press. 2016.

[38] Friedman JH. Greedy function approximation: A gradient boosting machine. https://doi.org/101214/aos/1013203451. 2001;29: 1189–1232. doi: 10.1214/AOS/1013203451

[39] Davis J, Goadrich M. The relationship between precision-recall and ROC curves. ACM International Conference Proceeding Series. 2006;148: 233–240. doi: 10.1145/1143844.1143874

[40] Tsoumakas G, Katakis I. Multi-label classification: An overview. International Journal of Data Warehousing and Mining. 2007. doi: 10.4018/jdwm.2007070101

[41] Jaccard P. THE DISTRIBUTION OF THE FLORA IN THE ALPINE ZONE. New Phytologist. 1912;11. doi: 10.1111/j.1469-8137.1912.tb05611.x

[42] Brown-Brandl T, Kapun A. Comparing three different passive RFID systems for behaviour monitoring in grow-finish pigs Cost efficient optimization of automatic lameness detection in dairy cattle tailored to the farmer View project Discrete Element Simulations to predict fruit bruising View project. 2017 [cited 2 Oct 2023]. Available: https://www.researchgate.net/publication/321974038

[43] Ni LM, Liu Y, Lau YC, Patil AP. LANDMARC: Indoor Location Sensing Using Active RFID. Wireless Networks. 2004;10: 701–710.

[44] Maselyne J, Adriaens I, Huybrechts T, De Ketelaere B, Millet S, Vangeyte J, et al. Measuring the drinking behaviour of individual pigs housed in group using radio frequency identification (RFID). Animal. 2016;10: 1557–1566. doi: 10.1017/S1751731115000774 25959418

[45] Jebali C, Kouki A. A Proposed Prototype for Cattle Monitoring System using RFID. 2018 International Flexible Electronics Technology Conference, IFETC 2018. 2018. doi: 10.1109/IFETC.2018.8583969

[46] Weis SA. RFID (Radio Frequency Identification): Principles and Applications. 2007.

[47] Krogius M, Haggenmiller A, Olson E. Flexible Layouts for Fiducial Tags. IEEE International Conference on Intelligent Robots and Systems. 2019; 1898–1903. doi: 10.1109/IROS40897.2019.8967787

